# Supplementary material for: Rapid genomic evolution in Brassica rapa with bumblebee selection in experimental evolution
Source: BMC Ecol Evol. 2024 Jan 9;24:7. doi: 10.1186/s12862-023-02194-y (PMC10775529; doi:10.1186/s12862-023-02194-y)
Supplement: Supplementary file 4 — Supplementary Material 4: Figure S1. Density of genetic markers for each 10 chromosomes. The x-axis represents the 10 Brassica rapa chromosomes. The y-axis represents the density of genetic markers. Figure S2. Distribution of filtered genomic data for our final dataset of 4’713 SNPs. (a) Distribution of the minor allele frequency, (b) distribution of average read depth (DP) per SNPs, (c) distribution of the average read depth (DP) per individual, (d) distribution of the average genotype quality (GQ), (e) different thresholds of the minimum average GQ as a function of the number of SNPs in the final dataset. The red line indicates the chosen value in our study (–minGQ = 15). Figure S3. Linkage disequilibrium and haplotype block structure. (A) Distribution of the median pairwise linkage disequilibrium (r2) for each population by distance between two SNPs (kb). (B) Number of haplotype block calculated within each population (C) Average length (kb) of haplotype blocks per population (more details Table S1) [file 12862_2023_2194_MOESM4_ESM.docx]

**Supplementary information**

**Title:** Rapid genomic evolution in *Brassica rapa* with bumblebee selection in experimental evolution

***Authors:***

Léa Frachon, Florian P. Schiestl

**Figure S1. Density of genetic markers for each 10 chromosomes.**


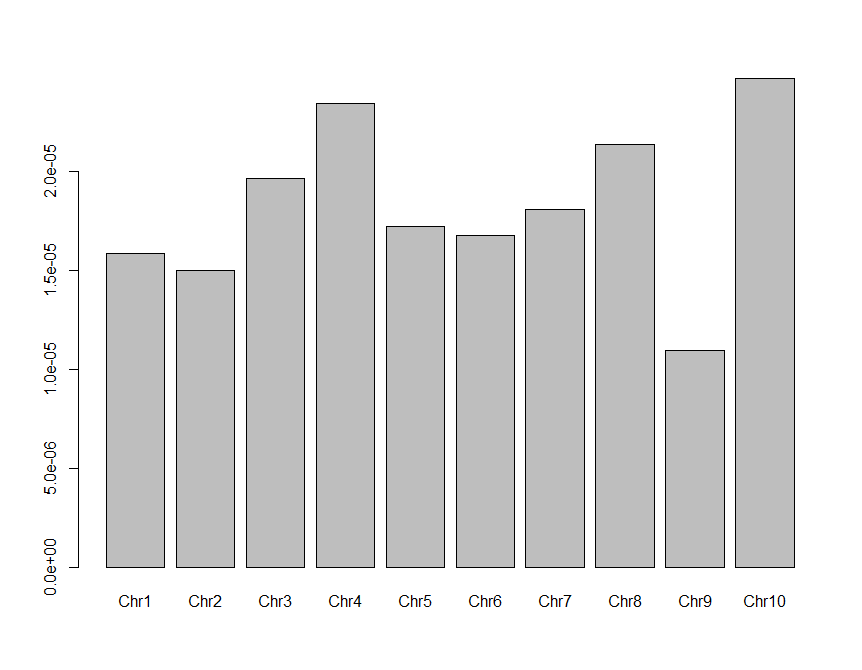


**Figure S2. Distribution of filtered genomic data for our final dataset of 4’713 SNPs. (a) Distribution of the minor allele frequency, (b) distribution of average read depth (DP) per SNPs, (c) distribution of the average read depth (DP) per individual, (d) distribution of the average genotype quality (GQ), (e) different thresholds of the minimum average GQ as a function of the number of SNPs in the final dataset. The red line indicates the chosen value in our study (--minGQ = 15).**

**
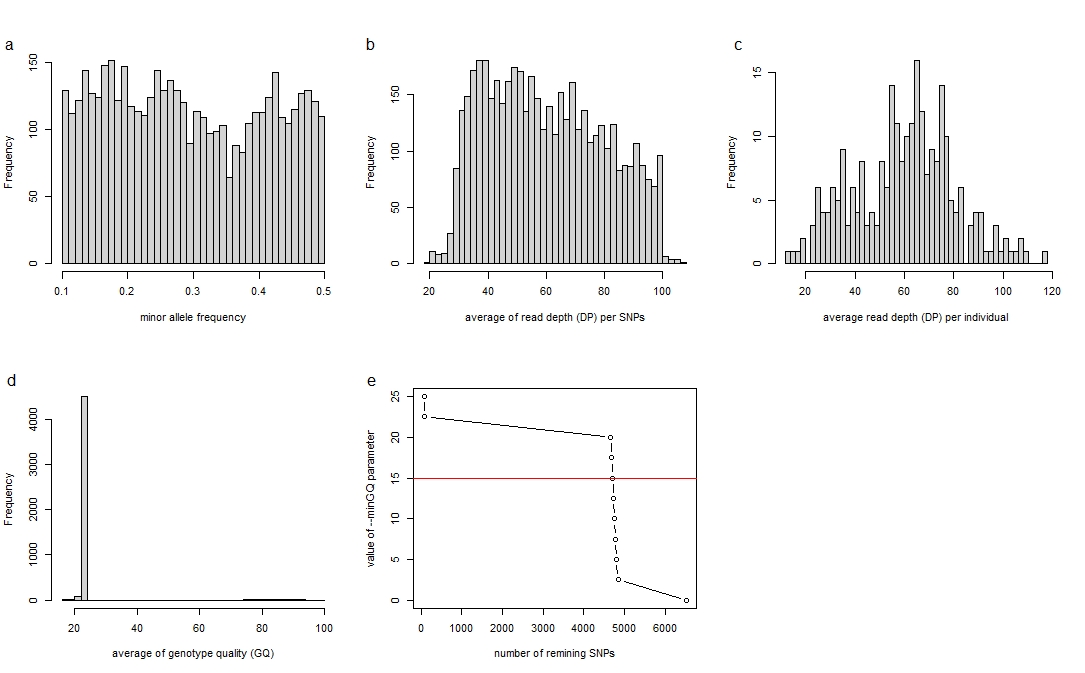
**

**Figure S3. Linkage disequilibrium and haplotype block structure. (A)** Distribution of the median pairwise linkage disequilibrium (r^2^) for each population by distance between two SNPs (kb). **(B)** Number of haplotype block calculated within each population **(C)** Average length (kb) of haplotype blocks per population (more details Table S1).

**
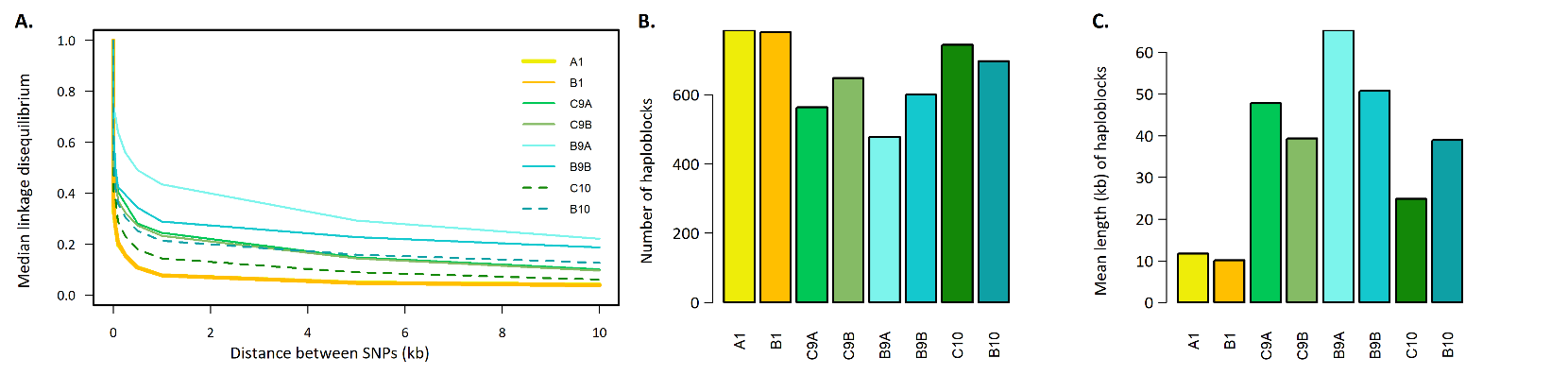
**

**Table S1. Haplotype blocks.** For each population, the number of haplotype blocks, the mean (+- sd) of the number of SNPs per haplotype block, and the length (in kb) of haplotype blocks.

**Table S2. Polygon area formed by six first principal component of genomic PCA.** The polygon areas were calculated for each 15 pairs of principal components explaining 56.07% of the total genomic variance. The areas were calculated for each population.

**Dataset1. List of candidate genes with significant allele frequency changes during bumblebee selection.** The transcripts are retrieved from phytozome.jgi.doe.gov.
